# The Role of Chronic Stress in the Pathogenesis of Ischemic Heart Disease in Women

**DOI:** 10.1002/cph4.70000

**Published:** 2025-02-04

**Authors:** Megan Cairns, Erna Marais, Danzil Joseph, M. Faadiel Essop

**Affiliations:** ^1^ Division of Medical Physiology Centre for Cardio‐Metabolic Research in Africa (CARMA) Faculty of Medicine and Health Sciences Stellenbosch University Cape Town South Africa; ^2^ Department of Physiological Sciences, Center for Cardio‐Metabolic Research in Africa (CARMA) Stellenbosch University Stellenbosch South Africa

**Keywords:** chronic stress, clinical, female, ischemic heart disease, preclinical

## Abstract

Psychological stress has emerged as a critical risk factor for cardiovascular disease, especially in women. While female participation in clinical research has improved, sex‐specific data analysis and reporting often remain inadequate, limiting our ability to draw definitive conclusions for women. Conversely, preclinical studies consistently demonstrate adverse effects of stress on female health, yet the molecular mechanisms underlying this association remain elusive. Evidence suggests that female IHD pathogenesis is more complex than in males, involving multiple factors, including inflammation, contractile dysfunction, bioenergetic impairment, and remodeling. However, many of these mechanisms are primarily derived from male studies, and molecular investigations in female models are limited, hindering our understanding of the underlying biological pathways. This is particularly concerning given the increasing prevalence of ischemic heart disease in postmenopausal women. In order to fully elucidate the impact of stress on female cardiac health and develop targeted interventions, further preclinical research on female models is essential.

## Graphical abstract

This review article focuses on the complex relationship between chronic stress and ischemic heart disease in females. The role of stress mediators in initiating cellular and molecular pathways in this context is highlighted.

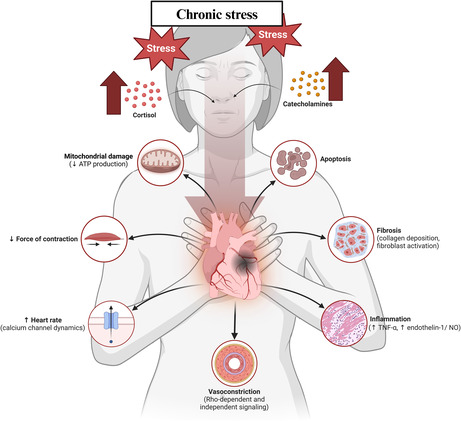

## 1 Introduction

**FIGURE 1:**
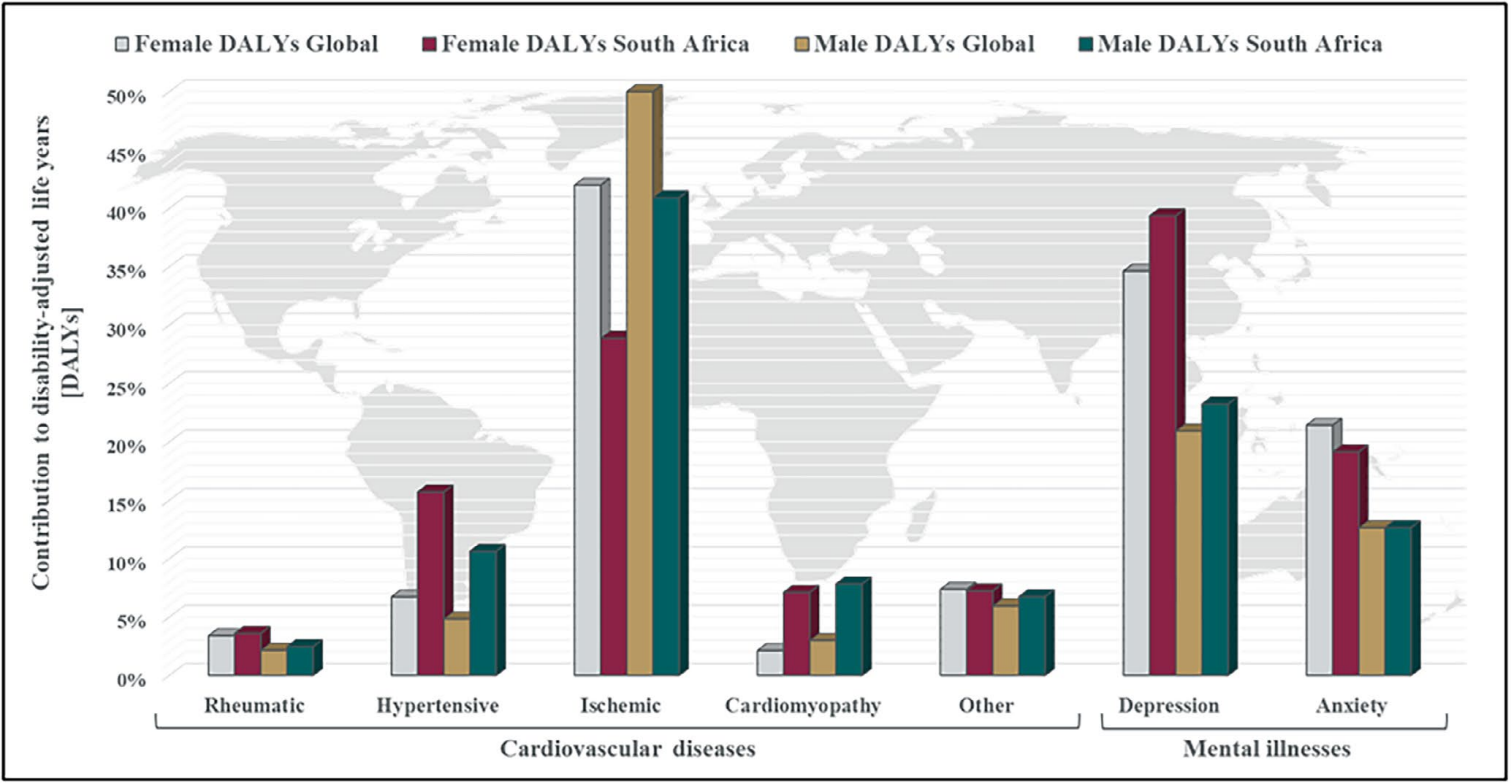
Male and female psychosocial stress‐related mental illness and cardio‐pathology disability‐adjusted life years (DALY) contribution to their respective non‐communicable diseases in 2019. Figure derived based on previously published data (Dattani et al. 2023; World Health Organization 2024).

Cardiovascular diseases (CVDs) are a leading global health concern (Figure 1), accounting for a significant proportion of noncommunicable disease deaths (World Health Organization (WHO) 2019). Ischemic heart disease (IHD) is a primary contributor to CVDs, influenced by a complex interplay of genetic and lifestyle factors. Psychological stress, including associated mental illnesses like depression and anxiety, has emerged as a modifiable risk factor for CVDs (Schneiderman, Ironson, and Siegel 2005; Sher et al. 2020; Momeni et al. 2019; Jimenez et al. 2019; American Psychological Association 2023). While the impact of psychosocial stress may seem relatively limited, with global prevalence rates estimated at around 4% and 3% for depressive and anxiety disorders, respectively, such complications affect millions of individuals worldwide (Institute for Health Metrics and Evaluation (IHME) 2018). The increasing demands of modern life, exacerbated by recent events like the COVID‐19 pandemic, contributed to a rise in stress‐related mental health challenges (Rana, Gulati, and Wadhwa 2019). Moreover, studies consistently show that individuals experiencing elevated stress, anxiety, or depression are at higher risk for developing CVDs (Al Omari et al. 2020; Iasevoli et al. 2021; Xiong et al. 2020).

Psychological conditions such as post‐traumatic stress disorder (PTSD), anxiety, and depression can contribute to CVD through a variety of mechanisms, including alterations to vascular tone, hemodynamics, inflammation, and metabolism (Brouillard et al. 2019; Duarte et al. 2015; Liu et al. 2015; Costa‐Ferreira et al. 2016; Firmino et al. 2019; Vieira et al. 2018; Chen et al. 2020; Shin et al. 2018; Perfilova et al. 2007; Şahin et al. 2006; Sood et al. 2006; Üresin et al. 2004; Gautam et al. 2015; Costoli et al. 2004). Conversely, the CVD event(s) itself may predispose such patients to develop psychological stress‐related mental illnesses (Birk et al. 2024; Grand et al. 2023). As such, these two conditions form a vicious cycle that promotes the incidence of further major adverse cardiac events (MACE) as supported by elevated odds ratios (Nyström et al. 2022; Sumner et al. 2015; Geulayov et al. 2018).

Despite the mounting evidence supporting the influence of chronic stress on CVD development, there are distinct challenges when determining their exact etiological connections: (1) a reliance on observational data, particularly retrospective and longitudinal studies; (2) complex interplay between chronic stress and CVD risk factors (e.g., diet and genetics); and (3) a lack of studies on the biological mechanisms underlying stress‐induced MACE (Schneiderman, Ironson, and Siegel 2005; Üresin et al. 2004; Mousavi et al. 2019). Specifically, there is uncertainty about whether stress is *actually* causing pathology, acting as a marker for other contributing factors (such as socioeconomic status), or occurring because of a previous cardiac event (e.g., acute myocardial infarction). Such a lack of insight may compromise the development of effective therapeutic interventions.

To further complicate matters, females are more likely to experience chronic stress and develop CVD, yet the vast majority of biological mechanistic information currently available is specific to males (Lopez and Bagot 2021). Males and females perceive, process, and respond very differently to stress, and a growing body of literature underscores the significant differences in CVD presentation, diagnosis, and pathology between the two sexes (Pravda et al. 2021; Aggarwal et al. 2018; Wright et al. 2023; Costa et al. 2021; Chaplin et al. 2008). Moreover, research indicates that women are more susceptible to stress‐induced myocardial infarction and experience worse cardiovascular outcomes despite less obstructive coronary artery disease (Reis et al. 2001; Garcia et al. 2016; Lichtman et al. 2018; DeFilippis et al. 2020; Vaccarino et al. 2018). These differences necessitate a sex‐specific approach to prevention and treatment strategies in cardiovascular health. While female inclusion in clinical studies has grown, there remains a significant gap in reporting female‐specific outcomes beyond generalized odds ratios (Merone et al. 2022). More recently, some preclinical studies took up this challenge as an attempt to mitigate this knowledge gap by incorporating females into their study design (Lopez and Bagot 2021). Such studies generally focus on the downstream effects of well‐known stress mediators such as cortisol and catecholamines, which affect various intracellular processes and pathways, including those involved in regulating cardiac function (Vieira et al. 2018; Vieira, Duarte, Costa‐Ferreira, and Crestani 2018; Moradi‐Kor et al. 2020).

This review will attempt to address this knowledge gap by systematically exploring the complex interplay between chronic psychological stress and IHD in females, by examining the clinical manifestations of stress‐related IHD and the downstream effects of stress mediators on cellular and molecular pathways. We aim to provide a comprehensive understanding of the potential risks associated with chronic stress and identify areas for future research and targeted interventions.

## 2 Psychosocial Stress

Stress is the generalized and non‐specific response (unique to both the individual and the nature of the stressor) to an allostatic load that threatens to disrupt homeostasis (Schneiderman, Ironson, and Siegel 2005; Selye 1983; Salleh 2008; Godoy et al. 2018; Kogler et al. 2015). Perceived stressors are broadly divided into two major categories—physical (e.g., infection or injury) and psychological (e.g., a negative work environment). This review focuses on psychological stress, defined as the emotional and physiological response to a perceived threat to an individual's psychological homeostasis (reviewed by James et al. (2023)). The degree of psychological stress experienced by an individual can be measured using three primary methods—self‐reporting (i.e., the Perceived Stress Scale); behavioral coding (i.e., the Trier Social Stress Test); and physiological markers (i.e., heart rate variability [HRV] and cortisol; Crosswell and Lockwood 2020).

Despite the limited availability of global psychological stress statistics, a recent Gallup survey (2023) offers valuable insights (GALLUP Inc 2024). Conducted across 122 countries, the survey assessed daily stress levels in employed people and revealed a significant increase from 33% in 2014 to a peak of 44% during the COVID‐19 pandemic. Although this figure has declined to 41%, it remains notably high. Globally, female and younger (≤ 35 years) employees reported experiencing more daily stress than their older or male counterparts. Six of the ten regions evaluated exceeded the global average, with the Middle East and North Africa, the United States and Canada, and sub‐Saharan Africa ranking highest at 52%, 49%, and 48%, respectively (GALLUP Inc 2024). While younger females tend to experience more stress on a global level, the situation in sub‐Saharan Africa is different, as older females (≥ 35 years) reported higher levels of daily stress (GALLUP Inc 2024).

Due to the highly individualistic perception of stress—even in the presence of standardized tests—it is relatively difficult to find an appropriate and accurate way to determine the actual degree of stress. A way around this is to study the prevalence and burden of diseases closely linked to stress, such as depression and anxiety. These psychological disorders share many similarities in terms of brain neuroendocrine system alterations that typically present in stressed individuals (Cui et al. 2024). For example, the American Psychological Association describes psychosocial stress as “a life situation that creates an unusual or intense level of stress that may contribute to the development or aggravation of mental disorder, illness, or maladaptive behavior” (American Psychological Association 2023).

Global data from 2021 indicate that anxiety and depressive disorders affect 4.4% and 4% of the population, respectively, and that these conditions primarily impact individuals aged 20–29 (Dattani et al. 2023). While African regions exhibit a similar age distribution, South Africa's prevalence rates for these conditions exceed global averages, with anxiety at 4.8% and depression at 5.3% (Dattani et al. 2023). Furthermore, global data indicate a gender disparity, with women experiencing depression at a rate of 4.8% compared to 3.2% in men. This disparity persists in Africa (5.9% vs. 4.2%) and South Africa (6.3% vs. 4.2%), suggesting that women in these regions are more vulnerable to these conditions. Notably, anxiety disorders follow a similar pattern, with higher prevalence among women globally, regionally, and in South Africa (Dattani et al. 2023).

### 2.1 Risk Factors

Psychological stress and associated mental conditions can be influenced by a variety of factors, including personal characteristics, environmental conditions, and societal features, which can interact and display high interpersonal variability (Martínez de Toda et al. 2019; Mofatteh 2021). Personality traits such as neuroticism or perfectionism predispose an individual to psychological stress, which can be further compounded by, or founded in, past trauma (Godoy et al. 2018; Rukh, de Ruijter, and Schiöth 2023). These traits and experiences may increase the adoption of ineffective and hazardous coping mechanisms, such as increased alcohol intake, as well as sleep deprivation and poor diet (Cho et al. 2020; Cabeza de Baca et al. 2019; Gibson 2006; Santosa et al. 2021). External factors such as poor work conditions and lack of social support structures, compounded by societal factors, can further impact stress levels (Ren et al. 2020; van der Molen et al. 2020).

Women are characterized as more “vulnerable” to stress, often exhibiting psychological symptoms in response to life stressors and reporting them more readily than men (Li and Graham 2017; Otten et al. 2021). They tend to internalize stress, leading more readily to mental disorders, while men are more likely to externalize stress through aggression and/or impulsivity (Eaton et al. 2012). Additionally, women experience unique risk factors due to biological and social factors. For example, they have a 6.5%–20% chance of developing postpartum depression and an elevated risk of developing postpartum psychosis if diagnosed with bipolar disorder (Liu, Wang, and Wang 2022; Sambataro et al. 2021; Friedman, Reed, and Ross 2023). Societal and cultural expectations involving various caregiving roles for children and elderly parents can also contribute to their stress levels (Li, Lee, and Lai 2022).

### 2.2 Stress Response

Although psychological and physical stressors initiate the same peripheral response through convergent signaling via the hippocampus, their central perception and processing differ (Godoy et al. 2018). For example, physical stressors activate the brain stem and hypothalamus to facilitate rapid systemic alterations, whereas psychological stressors are processed by the limbic system and contextualized by the prefrontal cortex. The peripheral stress response is primarily mediated by two primary physiological pathways, that is, the sympathetic‐adrenomedullary (SAM) system and the hypothalamic–pituitary–adrenal (HPA) axis.

**FIGURE 2:**
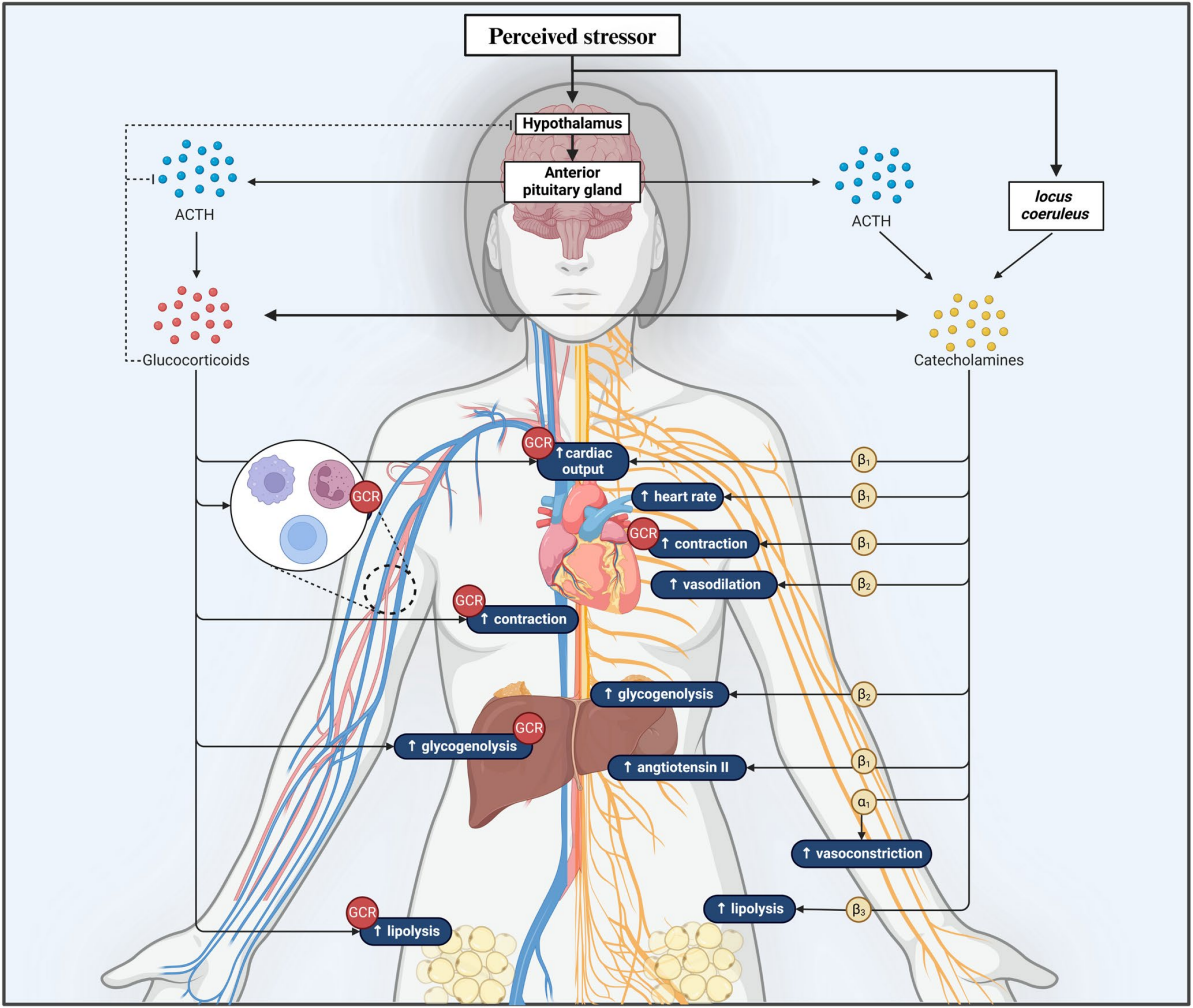
Physiological effects of the HPA axis and SAM pathway in response to acute, perceived stress. α_1_, adrenergic receptor alpha subtype 1; β_1_, adrenergic receptor beta subtype 1; β_2_, adrenergic receptor beta subtype 2; β_3_, adrenergic receptor beta subtype 3; ACTH, adrenocorticotropic hormone; GCR, glucocorticoid receptor. Created in BioRender.com.

The SAM system rapidly mobilizes the body for immediate action through the release of norepinephrine and epinephrine that can bind to α and β receptors based on ligand affinity (Tank and Wong 2015; Paravati, Rosani, and Warrington 2025; Motta E Motta et al. 2021). The collective interactions of norepinephrine and epinephrine on these receptors induce systemic effects aimed at priming the body for an acute stress response (Figure 2), including increased bioavailability of fuel substrates such as glucose and fatty acids, as well as elevated oxygen consumption and cardiac work (reviewed by Godoy et al. (2018)). In the coronary arteries, stimulation of β_2_ receptors on smooth muscle cells leads to vasodilation, enhancing perfusion and the delivery of oxygen and metabolites to the myocardium (Wu, Zeng, and Zhao 2021). Simultaneously, the release of norepinephrine from sympathetic nerve endings causes vasoconstriction in the systemic circulation via α_1_ receptors (Wu, Zeng, and Zhao 2021). This increase in peripheral resistance is offset by a compensatory rise in cardiac output, achieved through increased heart rate and contractility, both of which are also mediated by β_2_ receptor activation (Wu, Zeng, and Zhao 2021).

Conversely, the HPA axis initiates a more prolonged stress response involving the release of cortisol from the *zona fasciculata* of the adrenal cortex (Joseph and Golden 2017). Approximately 90%–95% of cortisol is bound to corticosterone‐binding globulin, effectively rendering it inactive (Timmermans, Souffriau, and Libert 2019). The remaining 5%–10% remains unbound and biologically active in circulation. As a steroid hormone, cortisol is lipophilic in nature and can passively diffuse across the cell membrane and subsequently bind to its intracellular receptors (Timmermans, Souffriau, and Libert 2019). The enzyme 11β‐hydroxysteroid dehydrogenase (11β‐HSD) regulates cortisol activity in peripheral tissues, with an increased 11β‐HSD1/11β‐HSD2 ratio potentiating or attenuating intracellular effects depending on the receptor target (Carranza‐Lira, Jimeno, and Ortiz 2023). Active intracellular cortisol can bind to mineralocorticoid and glucocorticoid receptors (GCR), influencing various genomic and non‐genomic processes (Pujols et al. 2002). Through such mechanisms, cortisol can elevate substrate availability, increase blood pressure, and reduce inflammation within an acute context (Schneiderman, Ironson, and Siegel 2005; Arlt and Stewart 2005). Negative feedback mechanisms involving such HPA axis mediators assist in maintaining homeostatic cortisol levels and prevent maladaptive signaling.

While the renin‐angiotensin‐aldosterone system (RAAS) is not traditionally categorized as a component of the stress response, its significant impact on blood pressure regulation and electrolyte balance underscores its systemic relevance (reviewed by Correa et al. (2021)). For example, catecholamines stimulate angiotensin II production, which can influence baroreflex responsiveness to fluctuations in blood pressure (Aldehni et al. 2011; Costa‐Ferreira, Gomes‐de‐Souza, and Crestani 2021; Firoozmand et al. 2018). The differential binding of angiotensin II to angiotensin receptor type 1 (ATR1) and type 2 (ATR2) elicits distinct physiological outcomes. Here, binding to ATR1 promotes vasoconstriction and aldosterone secretion, while ATR2 stimulation leads to vasodilation through nitric oxide (NO) release, along with anti‐inflammatory and anti‐proliferative effects (Correa et al. 2021). Conversely, persistent activation of ATR1 promotes inflammation, fibrosis, and cardiac hypertrophy, whereas ATR2 is implicated in apoptotic signaling (Firoozmand et al. 2018; Li, Li, and Yuan 2012). Although both receptor subtypes are expressed in adult tissues, angiotensin II exhibits a marked preference for ATR1, which is particularly abundant in vascular smooth muscle.

Although these pathways represent distinct branches of the physiological stress response, they do not function in isolation, with each system working in concert with the others to enhance or suppress activity and flux as required (Herman and Cullinan 1997; Sawchenko and Swanson 1982; Bjorklund and Hokfelt 1987; Pacák and Palkovits 2001; Reiche, Nunes, and Morimoto 2004; Goddard et al. 2010). However, prolonged exposure to a stressful stimulus alters the functionality of these systems and contributes to systemic pathology through maladaptive signaling.

### 2.3 Preclinical Pathophysiological Evidence

To assess the effects of psychological stress on the female heart, various animal models have been developed with specific behavioral and cardio‐metabolic endpoints. Tables 1, 2, 3 summarize the findings from these studies, encompassing anthropometric, cardiac, behavioral, hormonal, and intracellular data. While there is a general lack of consistency in the data collected, a striking observation is the dearth of molecular investigations. Although not exhaustive, these tables represent a significant portion of preclinical chronic stress research (especially rodents) focusing on the heart. However, only a few studies delve beyond hormonal and functional alterations to help contribute to our understanding of female CVD development in response to chronic psychological stress. These findings underscore the critical role of inflammation in female cardiac pathology (Brooks et al. 2018a, 2018b; Stanley et al. 2014).

**TABLE 1:** Preclinical rodent studies investigating the relationships between psychosocial stress and CVD in females (2000–2024).

Rodent model	Animal age	BW	Behavioral presentations		HPA/ SAM	Cardiovascular system							References
						Post‐intervention					Molecular markers		
			Depr.	Anxiety		BP	HR	Mass	Vasc. F	Overall function	Fibrosis	Inflam./OS	
Cycling													
Restraint stress (1–2 h, 10 days)	7–10 weeks	↓	↑	↑	↑	—	↑	—	↓	—			Vieira et al. (2018); Moradi‐Kor et al. (2020); Vieira, Duarte, Costa‐Ferreira, and Crestani (2018)
Restraint stress (1 h, 28 days)	10 weeks	—		—	↓	—	—			↓		↑	Cairns et al. (2024)
Overcrowding (2 weeks)	6.5 weeks					—	—	—	—	—			Ledvényiová‐Farkašová et al. (2015)
Cycling + SHR													
Restraint stress (1–2 h, 10 days)	10 weeks	↓			↑	—	↑	—	↓	↓			Vieira, Duarte, Costa‐Ferreira, and Crestani (2018)
Overcrowding (2 weeks)	6.5 weeks					—	—	—	↓	—			Ledvényiová‐Farkašová et al. (2015)
Non‐castrated males		↓	↑		↑	↑		↑		↓	↑	↑	Brouillard et al. (2019); Duarte et al. (2015); Liu et al. (2015); Costa‐Ferreira et al. (2016); Firmino et al. (2019); Vieira et al. (2018); Chen et al. (2020); Shin et al. (2018); Perfilova et al. (2007); Şahin et al. (2006); Sood et al. (2006); Üresin et al. (2004); Gautam et al. (2015); Costoli et al. (2004)

*Note:* The following table only includes in vivo models designed to mimic the effects of chronic stress, ranging from general daily stress to specific psychological stress‐related mental illnesses (i.e. depression, anxiety, or PTSD). All measurements were collected from adolescent or adult female rats.

Abbreviations: —, no change versus matched controls; ↑, significant increase versus matched controls; ↓, significant decrease versus matched controls; BP, blood pressure; BW, body weight; Depr., depression; HPA, hypothalamic–pituitary–adrenal axis hormones; HR, heart rate; Inflam., inflammation; OS, oxidative stress; RRS, repeated restraint stress; SAM, sympathetic adrenomedullary pathway hormones; SHR, spontaneously hypertensive rats; Vasc. F, vascular function.

A hallmark of stress research is to confirm successful induction of a chronic stress phenotype within the experimental cohort. This is typically determined through behavioral (i.e., elevated plus maze, forced swim, or open field tests) and/or hormonal (e.g., corticosterone) evaluations (Lee, Kim, and Choi 2015; Lezak, Missig Jr, and WAC. 2017). The majority of studies in Tables 1 and 2 reflect this, with increased circulating levels of corticosterone reported with depressive behaviors in cycling females when subjected to repeated restraint stress (RRS)/unpredictable chronic mild stress (UCMS)/chronic mild stress (CMS) models (Vieira et al. 2018; Vieira, Duarte, Costa‐Ferreira, and Crestani 2018; Moradi‐Kor et al. 2020; Brooks et al. 2018a, 2018b; Stanley et al. 2014). These alterations in adult females often coincide with diminished vascular function, yet do not appear to affect blood pressure or relative heart size (in proportion to body weight)—in contrast to their male counterparts (Brouillard et al. 2019; Duarte et al. 2015; Liu et al. 2015; Costa‐Ferreira et al. 2016; Firmino et al. 2019; Vieira et al. 2018; Chen et al. 2020; Shin et al. 2018; Perfilova et al. 2007; Şahin et al. 2006; Sood et al. 2006; Üresin et al. 2004; Gautam et al. 2015; Costoli et al. 2004). However, the combined effects of chronic stress and an obesogenic phenotype (i.e., obese Zucker rat (OZRs)) lower body weight and overall cardiac function while inducing cardiac hypertrophy, suggesting a predisposition for decompensated cardiomyopathy in such animals (Brooks et al. 2018b).

**TABLE 2:** A summary of preclinical studies investigating the relationships between depression and CVD in females (2000–2024).

Rodent model	Life stage	BW	Behavioral presentations		HPA/ SAM	Cardiovascular system							Ref.
						Post‐intervention					Molecular markers		
			Depr.	Anxiety		BP	HR	Mass	Vasc. F	Overall function	Fibrosis	Inflam./OS	
UCMS (8 weeks)													
Cycling	Adult	—	↑		↑	—			↓			↑	Brooks et al. (2018a); Brooks et al. (2018b); Stanley et al. (2014))
Cycling + MetS	Adult	↓	↑		↑	—		↑	↓	↓			Brooks et al. (2018b)
OVX	Adult	—	↑		—	—			↓			—	Brooks et al. (2018a); Brooks et al. (2018b)
OVX + MetS	Adult	—	↑		↑	—		↑	↓	↓			Brooks et al. (2018b)
CMS (7 weeks)													
Cycling + C57BL/6J		↓			—			↓					Terenina et al. (2019)
Cycling + C57BL/6NJ		↓			↑			↓					Terenina et al. (2019)
Cycling + DBA/2J		—			—			↓					Terenina et al. (2019)
Non‐castrated males		↓	↑	↑	↑	↑				↓	↑	↑	Firoozmand et al. (2018); Brooks et al. (2018a); Geng et al. (2020); Dang et al. (2016); Liu, Liu, and Zeng (2018); Hu et al. (2020); Matchkov et al. (2015); Wu et al. 2012; Grippo et al. (2004); Xinxing et al. (2014); Grippo, Moffitt, and Johnson (2008); Grippo et al. (2006); Grippo, Beltz, and Johnson (2003)

*Note:* The following table only includes in vivo models specifically designed to induce depressive symptoms. As such, only unpredictable chronic mild stress or chronic mild stress models were included.

Abbreviations: —, no change versus matched controls; ↑, significant increase versus matched controls; ↓, significant decrease versus matched controls; BP, blood pressure; BW, body weight; CMS, chronic mild stress; Depr., depression; HPA, hypothalamic–pituitary–adrenal axis hormones; HR, heart rate; Inflam., inflammation; MetS, metabolic syndrome; OS, oxidative stress; OVX, ovariectomized; Ref., reference; SAM, sympathetic adrenomedullary pathway hormones; UCMS, unpredictable chronic mild stress; Vasc. F, vascular function.

Despite consistent changes to circulating markers in cycling females, cardiovascular measurements are quite variable between the different models. For example, crowding stress failed to match the RRS model in terms of its cardiovascular effects (Ledvényiová‐Farkašová et al. 2015). This likely stems from a combination effect of the stressor type and intensity, as well as the age of the animals when subjected to chronic stress. Here, cycling females subjected to overcrowding stress were considered adolescent, i.e., between 6 and 7 weeks old, whereas RRS females were a bit older, i.e., between 7 and 10 weeks old. An additional consideration is the rodent strain employed in such studies, as genetics play a critical role in neuroendocrine and cardiovascular outcomes (Terenina et al. 2019).

Exposure to stress during pregnancy or postnatally can also influence cardiac function. A few rodent studies indicate increased anxiety, cardiac oxidative stress (maternal stress), and sex‐specific alterations to cardiovascular function with maternal separation (Arabi et al. 2021; Igosheva et al. 2004). Although the mechanisms underlying such changes still require further clarification, the evidence supports alterations to GCR expression and HPA axis responsiveness to stress (Duarte et al. 2015; Ladd et al. 2004). These effects are further amplified in the combination of maternal separation and chronic stress, where female Wistar rats showed increased neuroendocrine responses to maternal separation that persisted well into adulthood (Renard, Rivarola, and Suárez 2010; Aisa et al. 2008; Verma et al. 2022).

The presence of estrogen appears to be protective against the development of stress‐induced cardiac pathology under certain conditions. Surgically ovariectomized (OVX) rats effectively mimic the sex hormone deficiencies observed clinically in early post‐menopausal women and provide a useful preclinical model of this context. Here, OVX females display a lack of sex hormones together with elevated circulating glucose, cholesterol, and pro‐inflammatory cytokines—highlighting the baseline metabolic aberrations that promote CVD development (Brooks et al. 2018a). Of note, chronic stress exposure in the OVX females did not aggravate these metabolic or inflammatory aberrations, yet cycling UCMS females displayed increased circulating glucose and pro‐inflammatory cytokine levels despite no alterations to sex hormones. This suggests that the stress response itself can independently influence metabolic parameters outside of the sex hormones. However, these sex hormones do confer some degree of protection, as although cycling females subjected to the UCMS protocol displayed impaired vascular and cardiac function compared to unstressed controls, they still performed better than non‐castrated males or OVX females. Such females inherently possess greater estrogen levels, which assist in maintaining NO and prostacyclin levels, thus providing an additional layer of protection against chronic stress (Brooks et al. 2018a, 2018b).

Models of anxiety disorders provide far more comprehensive functional and molecular investigations into female preclinical stress research (Table 3). Exposure to chronic variable or chronic unpredictable stress models—regardless of the duration or genetic predisposition—significantly affects the body weight and circulating corticosterone levels of cycling adult females (Vieira et al. 2018; Vieira, Duarte, Costa‐Ferreira, and Crestani 2018; Roth et al. 2015). However, in the absence of genetic predisposition for hypertension or atherosclerosis, such females mimic the functional outcomes of the UCMS and RRS models—highlighting the female predisposition for vascular dysfunction over more traditional CVD risk factors such as elevated blood pressure (Vieira et al. 2018; Vieira, Duarte, Costa‐Ferreira, and Crestani 2018).

**TABLE 3:** A snapshot of preclinical studies investigating the relationships between anxiety disorders and CVD in females and males (2000–2024).

Rodent model	Life stage	BW	Behavioral presentations		HPA/ SAM	Cardiovascular system								Ref.
						Post‐intervention					Molecular markers			
			Depr.	Anxiety		BP	HR	Mass	Vasc. F	Overall function	ANP/BNP	Fibrosis	Inflam.	
Cycling														
PE + SS (1 h + 31 days)	Adult	—	—	↑						—				Rorabaugh et al. (2015); Zoladz et al. (2019)
TW (10 min, 5 days)	Adolescent				↑		↑	↑						Mousavi et al. (2019)
ISO (5 mg/kg/day, 10 days)	Adult	—			↑	—	—	↑		—	—	↑	—	Hou et al. (2021)
ISO (10 mg/kg/day, 14 days)	Adult	—			↑		↓	—			—		—	Ndzie Noah et al. (2023)
CUS/CVS (10 days)	Adult	↓		↑	↑	—	—	↑	↓	—				Vieira et al. (2018); Vieira, Duarte, Costa‐Ferreira, and Crestani (2018)
Cycling + SHR														
CUS (10 days)	Adult	↓			↑	—	—	—	—					Vieira, Duarte, Costa‐Ferreira, and Crestani (2018)
Cycling + ApoE^−/−^ Fbn1^C1039Gþ+/−^														
CVS/CUS (6 h, 5 days)	Adult	↓			↑					↓				Roth et al. (2015)
OVX														
PE + SS (1 h + 31 days)	Adult			↑										Zoladz et al. (2019)
ISO (5 mg/kg/day, 10 days)	Adult	—			↑	↓	↓	↑		↓	↑	↑	↑	Hou et al. (2021)
ISO (10 mg/kg/day, 14 days)	Adult	—			↑		↓	↑			↑		↑	Ndzie Noah et al. (2023)
OVX + E2														
ISO (5 mg/kg/day, 10 days)	Adult	—			↑	—	—	↑		—	—	↑	—	Hou et al. (2021)
ISO (10 mg/kg/day, 14 days)	Adult	—			↑		↓	↑			↑		↑	Ndzie Noah et al. (2023)
Non‐castrated males		↓	↓	↑	↑	↑		↑		↓		↑	↑	Duarte et al. (2015); Costa‐Ferreira et al. (2016); Firmino et al. (2019); Vieira et al. (2018); Mousavi et al. (2019); Vieira, Duarte, Costa‐Ferreira, and Crestani (2018); Rorabaugh et al. (2015); Kumari et al. (2003); Romic et al. 2020; Simas et al. (2018); Cudnoch‐Jedrzejewska et al. (2015); Schaeuble et al. (2019); Goodson et al. (2017); Cruz et al. (2016); Djordjevic et al. (2012)

*Note:* The following table only includes in vivo models specifically designed to induce anxiety disorders (i.e. anxiety, or PTSD). As such, only trauma witness, isoproterenol treatment, predator exposure, chronic variable stress, chronic unpredictable stress, and social stress models were included.

Abbreviations: —, no change versus matched controls; ↑, significant increase versus matched controls; ↓, significant decrease versus matched controls; ANP/BNP, atrial/brain natriuretic peptide; BP, blood pressure; BW, body weight; CUS, chronic unpredictable stress; CVS, chronic variable stress; Depr., depression; E2, estradiol; HPA, hypothalamic–pituitary–adrenal axis hormones; HR, heart rate; Inflam., inflammation; ISO, isoproterenol; OVX, ovariectomized; PE, predator exposure; Ref., reference; SAM, sympathetic adrenomedullary pathway hormones; SS, social stress; TW, trauma witness; Vasc. F, vascular function.

While chronic variable or unpredictable stress models utilize a variety of stressors applied in a randomized, unpredictable manner to induce alterations to the HPA axis that mimic anxiety disorders, other models focus instead on perturbations of catecholamine levels. For example, daily injections of isoproterenol (a sympathomimetic amine simulating both norepinephrine and epinephrine) facilitate increased heart rate and contractility through interactions with β receptors (Ma et al. 2020). However, OVX females and those supplemented with estradiol did not exhibit the same heart rate response as cycling female Sprague–Dawley rats or FVB mice (Hou et al. 2021; Ndzie Noah et al. 2023). While all three groups exhibited cardiac hypertrophy in response to a relatively mild isoproterenol dose, the OVX group demonstrated an exacerbated hypertrophic response with concurrent increases in atrial and B‐type natriuretic peptides and cardiac fibrosis. This group also exhibited a proinflammatory phenotype within the myocardium together with upregulated inducible NO synthase (iNOS) peptide levels. While estradiol supplementation partially protected OVX rats from isoproterenol‐induced cardiac damage, it was not sufficient to completely reverse cardiac impairment.

While isoproterenol administration offers a valuable tool for understanding the mechanistic underpinnings of anxiety‐induced CVD, its limitations should be considered. As anxiety disorders are multifaceted conditions that extend beyond elevated catecholamines, isoproterenol studies often neglect to examine the impact of repeated administration on other stress systems such as the HPA axis, which plays a prominent role during chronic anxiety. Of note, predator exposure or trauma witness models, often supplemented with daily stressors, provide a more comprehensive representation of anxiety disorders (Mousavi et al. 2019; Rorabaugh et al. 2015; Zoladz et al. 2019). However, such models frequently lack detailed cardiovascular and neuroendocrine measurements, which hinders our ability to identify female‐specific mechanisms or presentations of anxiety‐related CVD.

While such preclinical studies emphasize the role of chronic stress in exacerbating female cardiac dysfunction, they also highlight a significant gap in understanding the molecular and cellular mechanisms involved. This lack of molecular insight becomes particularly relevant when considering IHD, a leading cause of disease in especially postmenopausal women (World Health Organization 2024; Aggarwal et al. 2018). Thus, understanding the interplay between stress responses and IHD is essential for developing effective therapeutic strategies for female patients suffering from such interrelated cardiovascular complications.

## 3 Ischemic Heart Disease: A Female Perspective

Ischemic heart disease is a broad term encompassing cardiovascular conditions characterized by insufficient oxygenated blood supply to the heart, caused by narrowed or blocked coronary arteries (reviewed by Jensen, Hjortbak, and Bøtker (2020)). IHD is also therefore interchangeably referred to as coronary heart disease, coronary artery disease, or myocardial ischemia and includes a range of cardiac pathologies such as stable and unstable angina, as well as myocardial infarction (Jensen, Hjortbak, and Bøtker 2020). Although the disease burden is similar between the two sexes, risk factors, clinical presentation, and pathogenesis do show differences (Aggarwal et al. 2018). Here, pregnancy (e.g., pre‐eclampsia), hormonal fluctuations (e.g., menopause), as well as malignancies all compound the traditional risk factors that contribute to CVD development (Pravda et al. 2021; Cho et al. 2020). The diagnosis can also be more challenging as females often present with “atypical symptoms” such as fatigue, dyspnea, anorexia, presyncope, weakness, and pain not exclusive to the chest during unstable angina (Pravda et al. 2021; Aggarwal et al. 2018; Bracey and Brown 2008; Bairey Merz et al. 2006; Canto 2007; Khan et al. 2013). These symptoms are often dismissed as “non‐cardiac related” by both patients and medical practitioners alike, which contributes to misdiagnosis, delays effective treatment, and negatively affects prognosis (Pravda et al. 2021; Lichtman et al. 2018; Kirchberger et al. 2012). Evidence suggests that female IHD pathogenesis is far more multifactorial than that of their male counterparts due to the more prominent roles of microvascular and endothelial dysfunction, as well as inflammation (Aggarwal et al. 2018; Bairey Merz et al. 2006; Quyyumi 2006). This differential pathophysiology of IHD is influenced by several factors, including hormonal and anatomical differences. Male patients typically experience disordered blood flow and low shear stress, conditions that contribute to the formation of focused, calcified atherosclerotic plaques within their larger coronary arteries (Patel et al. 2016). These plaques are prone to rupture, leading to acute coronary syndromes. In contrast, females tend to have smaller coronary arteries with higher blood flow and endothelial shear stress, which can reduce inflammation and thrombosis and promote a more stable, diffuse, non‐calcified atherosclerotic pattern (Bairey Merz et al. 2006; Patel et al. 2016; Nugent, Mehta, and Bairey Merz 2011). However, a decline in estrogen levels can lead to endothelial dysfunction, characterized by impaired nitric oxide production and increased inflammation (reviewed by Raj et al. (2023)).

The IHD subclasses collectively accounted for almost half of all CVD‐related deaths worldwide in 2019 and display regional differences in mortality (World Heart Federation 2023). The World Health Organization estimates that IHD contributes to 49% of CVD‐related global deaths in women in developed nations such as the United States (World Health Organization 2024). However, IHD is less prevalent in South Africa, where it accounts for 26% of CVD‐related deaths. Regardless of socioeconomic status, age is a major contributor to the prevalence of IHD. South African pre‐menopausal women (30–49 age group) display a relatively lower risk of IHD‐related death compared to their United States counterparts (25% vs. 36%). This trend continues into the post‐menopausal phase (50–59 age group) where the disparity becomes even more pronounced (27% vs. 50%). South African women may not be exposed to the same lifestyle‐related risk factors as their American counterparts, which may explain their lowered IHD prevalence. Conversely, this may be attributed to potential underdiagnosis of IHD in South Africa due to healthcare access disparities or simply due to underreporting.

Ischemic heart disease is influenced by a complex interplay of non‐modifiable and modifiable risk factors. Non‐modifiable factors include genetics, sex, and advancing age (Kasprzyk et al. 2018). The latter two factors generally coincide with an elevated risk of IHD, with men experiencing a relatively higher risk at earlier ages than women. However, the risk for women increases significantly after menopause (reviewed by Ryczkowska et al. (2023)). Genetic factors cannot be overlooked, particularly in the context of depression and IHD. For example, two studies investigated the genetic vulnerability of both depression and risk for IHD in Vietnamese and Swedish twin populations (Xian et al. 2010; Kendler et al. 2009). Overall, these studies confirmed that a high genetic risk for depression increased the incidence of IHD, particularly in women. Cho et al. (2020) extensively reviewed the sex‐related risk factors for CVD in women and considered various contributors such as the use of perimenopausal hormone therapy and psychosocial issues. Here, female‐specific socioeconomic hormonal, and psychological risk factors compounded the physiological disturbances induced by traditional factors and primarily facilitated CVD onset via inflammatory mechanisms.

Conversely, modifiable factors, such as smoking, hyperglycemia, hypertension, hypercholesterolemia, obesity, sedentary behavior, and chronic psychological stress, can be altered through lifestyle changes and/or therapeutic interventions (Kasprzyk et al. 2018). Notably, chronic stress often intersects with other IHD risk factors, such as poor diet, sleep quality, and alcohol consumption (Cho et al. 2020; Cabeza de Baca et al. 2019; Gibson 2006; Santosa et al. 2021). Substantial evidence supports the relationship between psychosocial stress and cardiovascular complications (Sumner et al. 2015; Xian et al. 2010; Ebrahimi et al. 2021; Cho et al. 2015; Carranza‐Lira, Jimeno, and Ortiz 2023; Jee et al. 2019; Rutledge et al. 2014). For example, the INTERHEART study focused on the relationship between modifiable risk factors (e.g., psychological stress, smoking, hypertension, diabetes, physical activity, consumption of alcohol) for acute myocardial infarction at a global level (Rosengren et al. 2004; Yusuf et al. 2004). Here, stressed individuals were at a greater risk for myocardial infarction, particularly in men subjected to work‐related stressors (Rosengren et al. 2004).

The existing literature investigating the association between psychological stress‐related mental illnesses and IHD primarily relies on retrospective, observational, and longitudinal studies. Table 4 includes a selection of clinical studies reporting on the relationship between psychosocial stress‐related mental illnesses and IHD in females between 2000 and 2024. Of note, most studies tabulated here exclusively focus on the association between mental illness and IHD through the lens of odds ratios, neglecting the direct measurement of cardiovascular function or systemic health indicators, such as hormone levels or inflammatory markers. Despite increased female participation in clinical research, sex‐specific data analysis and reporting often remain inadequate, hindering the ability to draw definitive conclusions for women. In agreement, a search of current literature revealed numerous studies including females in the sample size but still failed to report on female‐specific outcomes (Boyle et al. 2013; Moazzami et al. 2023; Cohen et al. 2009). While some studies attempted to address this by presenting data for both sexes, comparisons are frequently limited to inter‐sex differences and lack a control group for non‐diseased individuals (Geulayov et al. 2018; Vaccarino et al. 2018). While such studies offer valuable sex‐specific odds ratios, they are relatively limited in their ability to elucidate female‐specific risk factors and the underlying mechanisms for IHD for those suffering from mental illnesses. Furthermore, most studies focused on specific cardiovascular functional parameters (e.g., blood pressure and HRV) without a comprehensive assessment of suitable biomarkers.

**TABLE 4:** Clinical studies reporting on the relationship between psychosocial stress‐related mental illnesses and IHD in females (2000–2023).

Mental illness	Study design	Associated condition	Cohort description	MACE hazard ratio	Clinical characteristics				Cardiovascular Parameters					Ref
					EGC abnormalities	CAD severity	IMT	Inflam.	BP	HR/HRV	RPP	End. F	AS	
Depression	Prospective	CAD^a^	Men and women ≥ 60 years	↑										Guimarães et al. (2017); Vaccarino et al. (2007)
	Cross‐sectional	MI^a^	Post‐MI patients 50 years	↑	—	↓			↑	↑	↑	—	—	Vaccarino et al. (2018)
	Prospective/Cross‐sectional	ASCVD	Women ~53 years	↑			↑							Carranza‐Lira, Jimeno, and Ortiz (2023); Jee et al. (2019)
	Cross‐sectional	MI^a^	Men and women Middle age	↑					—					Nyström et al. (2022)
	Retrospective/Observational	MI	Women 25–64 years	↑										Rukh, de Ruijter, and Schiöth (2023); Gafarov et al. (2013)
	Prospective	IHD	Women 58 years	↑										Rutledge et al. (2014)
	Longitudinal	Unspecified^a^	Men and women 66 years	↑										Geulayov et al. (2018)
	Cross‐sectional	CAD	Women 61 years		↑				↑		—			Cho et al. (2015)
PTSD	Prospective	MI	Women ~ 52 years	↑										Sumner et al. (2015)
	Retrospective/Longitudinal	CAD/MI	Women ~40 years	↑										Ebrahimi et al. (2021); Ebrahimi, Dennis, Shroyer, et al. (2024)
PTSD	Longitudinal	TIA	Women ~40 years	↑										Ebrahimi, Dennis, Alvarez, et al. (2024)
	Observational	ASCVD	Men and women 26 years	↑						↓				Holmstrup et al. (2020)
	Cross‐sectional	Unspecified	Women ~28 years						↑	↓			↑	Ahmed et al. (2024)
Anxiety	Observational	CAD	Women 50.6 years	↑					↑			↓	↑	Sara et al. (2021)
	Cross‐sectional	CAD	Women 55 years	↑										Abbasi et al. (2016)
	Longitudinal	Unspecified^a^	Men and women 66 years	↑										Geulayov et al. (2018)
	Prospective	MI	Women ~60 years	↑										Parkin et al. (2023)

Abbreviations: —, no change versus matched controls; ↑, significant increase versus matched controls; ↓, significant decrease versus matched controls; AS, arterial stiffness (includes pulsatility index and vessel diameter response to acetylcholine); ASCVD, atherosclerotic cardiovascular disease; BP, blood pressure; CAD, coronary artery disease; ECG, electrocardiogram; End. F, endothelial function (includes reactive hyperemia index, flow‐mediated dilation, and change in flood flow in response to acetylcholine); HR/HRV, heart rate/heart rate variability; IHD, ischemic heart disease; IMT, intima‐media thickness; MACE, major adverse cardiac event; MI, myocardial infarction; PTSD, post‐traumatic stress disorder; RPP, rate pressure product; TIA, transient ischemic attack.

^a^ Versus males of the same condition.

A notable limitation of current clinical research is the reliance on questionnaire scores and self‐reporting to assess psychological stress‐related mental illnesses. Such methods can introduce bias and may not accurately capture the underlying physiological changes associated with such conditions. For example, a patient may experience psychological stress but may not present with classical behavioral symptoms, or they may not perceive that they do (Haberer, Trabin, and Klinkman 2013; Davies et al. 2022). It is therefore essential to consider—especially in females—the role of hormonal influences such as cortisol, catecholamines, and other steroid hormones in the pathogenesis of IHD. It is our opinion that the exploration of inflammatory markers could provide valuable insights into mechanisms underlying this association, as this is characteristic of female IHD pathogenesis (Bairey Merz et al. 2006). Thus, by incorporating a wider range of cardiovascular parameters and biomarkers, future studies should be able to provide a more nuanced understanding of the mechanisms whereby psychological stress‐related mental illnesses contribute to the onset and development of IHD.

As highlighted in Table 4, HRV is the most frequently measured cardiovascular parameter in clinical studies and reflects the variation in time between successive heartbeats, a metric controlled by the autonomic nervous system (Cribbet et al. 2020). A reduced HRV suggests an imbalance in the autonomic nervous system, with a predominance of sympathetic nervous system activity over parasympathetic activity (Yugar et al. 2023). Psychological stress is a well‐documented factor influencing this imbalance, and preclinical investigations into general psychological stress or anxiety disorders in rodents consistently demonstrate elevated heart rates (Mousavi et al. 2019; Vieira, Duarte, Costa‐Ferreira, and Crestani 2018). An overactive sympathetic nervous system can manifest as both decreased HRV and an increased resting heart rate.

Evidence suggests that women are more susceptible to developing PTSD following certain types of trauma, such as child abuse and sexual assault (van der Kolk 2000). Moreover, the presence of trauma (regardless of a PTSD diagnosis) is a significant risk factor for IHD (Sumner et al. 2015). Notably, a diagnosis of PTSD following initial MACE increases the risk of future cardiovascular complications, such as Takotsubo cardiomyopathy (Primus and Auer 2011; Salmoirago‐Blotcher et al. 2016; Harb et al. 2015). Emotional triggers can also act as a tipping point, exacerbating the allostatic load imposed by other mental or physical health conditions and leading to the development of additional cardiac pathology (Princip et al. 2022).

A key observation from Table 4 is that almost all studies listed include post‐menopausal women. The higher risk for IHD‐associated MACE in post‐menopausal women is generally attributed to the lack of estrogen (Aggarwal et al. 2018). However, some studies in younger individuals highlight an increased risk for IHD in women < 40 years old, particularly those presenting with PTSD symptoms (Ebrahimi et al. 2021; Holmstrup et al. 2020). Such women often present with increased arterial stiffness and lowered HRV, which coincides with an elevated risk for MACE (Holmstrup et al. 2020; Ahmed et al. 2024). This suggests that such women lack sufficient cardioprotection despite relatively intact estrogen levels. Recent studies now suggest that progesterone (and not estrogen) displays an association with CVD in pre‐menopausal women (Chen et al. 2024). These findings indicate that other factors (not always considered) likely play a role in influencing the data generated.

Psychological stress is consistently linked to elevated corticosterone levels in preclinical models, irrespective of the chronic stress paradigms employed (as summarized in Tables 1, 2, 3). Prolonged exposure to heightened corticosterone can lead to maladaptive stress responses, contributing to the development of cardiovascular dysfunction. Of note, although IHD exhibits analogous functional impairments, clinical investigations still need to comprehensively explore mechanisms that connect psychological stress to IHD.

## 4 Psychological Stress and Female IHD

Most clinical research thus far has focused on establishing a correlation between these two conditions rather than elucidating the underlying pathways. Ebong et al. (2024) highlight the prominent role of vasomotor reactivity, microvascular dysfunction, and inflammation in female CVD development and emphasize the influence of psychological stress on these parameters. As such, this section will examine how a maladaptive stress response may influence the classical features of female IHD by considering the downstream effects of activation of stress response pathways in this context by focusing on microvasculature and cardiomyocyte dysfunction. During this process, we will also consider data generated in male in vivo research studies and in vitro experiments because of the lack of studies completed using females.

### 4.1 SAM Pathway

Previous research demonstrated that lowered HRV and elevated heart rate often reflect heightened sympathetic nervous system activity, a common marker of psychological stress (Table 4). Such hyperactivation triggers the release of norepinephrine, which binds to β1‐adrenergic receptors on cardiomyocytes to thereby induce both chronotropic and inotropic effects. The subsequent increase in intracellular cyclic adenosine monophosphate (cAMP) activates protein kinase A (PKA), which can phosphorylate various proteins involved in cardiac contractility, including L‐type calcium channels and ryanodine receptors (Liao et al. 2010). Augmented phosphorylation of such proteins increases their open‐state probability and calcium influx during depolarization, which is essential for contraction (Petrovic et al. 2008). However, excessive PKA activity increases Jun N‐terminal kinase phosphorylation and Ca^2+^/calmodulin‐dependent protein kinase II activity, which collectively facilitate intrinsic apoptosis (Wang et al. 2015).

Norepinephrine exerts a dual effect on the Rho/ROCK pathway, eliciting distinct outcomes based on the specific receptor subtype it binds (Sanada et al. 2004; Qiao, Huang, and Lum 2003). For example, β1 receptor activation on cardiomyocytes increases cAMP/PKA activity and facilitates hypercontractility through direct effects on calcium handling proteins (Hong, Ji, and Lai 2021). The Rho/ROCK kinase pathway regulates various cellular functions of vascular smooth muscle cells, including contraction, motility, proliferation, and differentiation; it is a key mechanism (Shimokawa, Sunamura, and Satoh 2016). It is implicated in the development of cardiomyopathy due to the pathway's established influence on vascular function and also as a result of its proapoptotic, fibrotic, and hypertrophic signaling within cardiomyocytes (Surma, Wei, and Shi 2011). While research studies explored the link between psychological stress and IHD, the specific role of the Rho/ROCK kinase pathway in this context remains unclear. cAMP/PKA pathway is a well‐known inhibitor of RhoA signaling, which represents a paradoxical situation as the observed upregulation of Rho/ROCK activity results in microvascular dysfunction. It's crucial to recognize that the effects of cAMP/PKA signaling on Rho/ROCK are highly dependent on the specific adrenergic receptor subtype and cell type. Alpha receptors on smooth muscle cells often bypass PKA and instead activate phospholipase C or couple to Gi proteins (Hong, Ji, and Lai 2021). This alternative signaling pathway results in decreased cAMP levels and a subsequent increase in Rho/ROCK activity. In support, hypercortisolemia promotes coronary vasospasm via increased Rho‐kinase activation (Hizume et al. 2006).

Concomitant with increased heart rate and attenuated HRV, elevated shear stress on blood vessels can contribute to the increased arterial stiffness observed in clinical settings (Table 4). Under normal physiological conditions, endothelial cells respond to elevated shear stress by producing NO, which induces vasodilation and lowers shear stress (Ando and Yamamoto 2022). However, there is an attenuation of NO production under pathophysiological conditions with abundant pro‐inflammatory cytokines (Münzel et al. 2017). Endothelin‐1 is a vasoconstrictor released by endothelial cells and exhibits an antagonistic relationship with NO (Mudau et al. 2012). Its production is stimulated by elevated shear stress, hypoxia, inflammatory cytokines, and angiotensin‐II. A shift towards increased endothelin‐1 production relative to NO can lead to inadequate vasodilation and persistent vasoconstriction to further aggravating shear stress and thereby contributing to microvascular dysfunction (Bakker et al. 2002).

Increased heart rate means that there will be greater energetic demands to ensure cardiac contractility is optimally sustained. Mitochondria are the primary source of ATP in highly metabolically active cells like cardiomyocytes and are influenced by cortisol in a bi‐phasic manner. Here, physiological levels promote oxidative phosphorylation and maintenance of the mitochondrial membrane potential, while supraphysiological levels can induce dysfunction (Dang et al. 2016). Picard and McEwen (2018) provide a detailed review of psychological stress and mitochondrial dynamics across numerous tissue and cell types. For example, chronic stress exposure can induce cardiac mitochondrial swelling and vacuolization, cause the loss of cristae integrity, and trigger impaired coupling efficiency and ATP synthase activity, which collectively culminate in diminished energy production (Liu et al. 2004; Soldani et al. 1997; Gesi et al. 2002). Both the power stroke and detachment phases of the myosin‐actin cross‐bridge cycle require ATP, which, despite increased calcium availability due to PKA activation, may not be able to occur under chronic hypercortisolemic conditions (Sugi, Akimoto, and Chaen 2018).

While a pro‐inflammatory state is commonly linked to hypercortisolemia and diminished GCR sensitivity, the influence of catecholamines in the progression of IHD warrants consideration (Amasi‐Hartoonian et al. 2022). Recent advancements in real‐time intravital imaging highlight the time‐dependent effects of chronic stress in adult male mice subject to RRS, with enhanced myeloid cell infiltration, rolling, and adhesion within the microvasculature in BALB/c and ApoE^−/−^ strains (Jang et al. 2024). While these effects are not solely attributed to catecholamines, previous research implicates norepinephrine in inflammatory leukocyte accumulation within cardiac and vascular tissues, with the latter contributing to atherosclerotic plaque development during acute stress (Hinterdobler et al. 2021). Female mice subjected to chronic variable stress demonstrated increased adrenergic stimulation of β_3_ receptors, resulting in hematopoietic stem cell proliferation and a corresponding elevation of circulating inflammatory monocytes, leukocytes (e.g., CD8+ T cells), and neutrophils (Heidt et al. 2014; Dolfi et al. 2024). This phenomenon is also observed clinically, where heightened perceived stress occurs in parallel with increased circulating leukocyte counts—provided leukocytes maintain GCR sensitivity (Heidt et al. 2014; Cohen et al. 2012).

### 4.2 HPA Axis

As previously discussed, cortisol is primarily anti‐inflammatory in terms of its function. Under acute stress conditions, it can bind to its GCR and facilitate transrepression of nuclear factor kappa‐light‐chain‐enhancer of activated B cells (NFκB) gene transcription and its associated production of pro‐inflammatory cytokines (Petta et al. 2016). However, it remains unclear whether GCR undergoes decreased/increased sensitivity or decreased/increased expression during chronic stress. The general consensus is that there is a concurrent rise in peripheral tissue receptor levels under conditions of hypercortisolemia (Ladd et al. 2004; Teles et al. 2013). However, alternative splicing and receptor isoform variations complicate matters, as the α isoform exhibits cortisol binding affinity, while the β isoform exerts dominant‐negative effects on the former (Merkulov and Merkulova 2012; Yudt et al. 2003). Although the α isoform is generally more abundant across various tissues under normal physiological conditions, β isoform levels can increase in response to inflammatory or stress stimuli and contribute to glucocorticoid resistance (Derijk et al. 2001). Thus, tissue‐specific α/β isoform and 11β‐HSD1/ 11β‐HSD2 ratios determine whether the higher circulating levels of corticosterone translate to increased intracellular actions. The anti‐inflammatory effects of cortisol are effectively attenuated under chronic stress conditions either due to increased intracellular inactivation by 11β‐HSD2 or because of the lack of receptor binding. Increased levels of pro‐inflammatory cytokines such as interleukin‐6 and tumor necrosis factor α (TNF‐α) can directly affect cardiac function by promoting inflammation within the myocardium, leading to greater oxidative stress and further cardiac damage (comprehensively reviewed by Rolski and Błyszczuk (2020)).

There was elevated cardiac PKA activity in male Wistar rats subjected to a severe model of chronic restraint stress, that is., 6 h daily over a period of 3 weeks (Wang et al. 2012). Interestingly, increased PKA activity correlated with enhanced phosphorylation and translocation of nerve growth factor‐induced clone B from the nucleus to mitochondria, thereby potentiating intrinsic apoptotic signaling (Wang et al. 2012). Additional studies utilizing a UCMS model of depression confirmed increased neuregulin‐1, Bax, and pErbB4 levels in combination with suppressed Akt/ERK phosphorylation within the myocardium, providing mechanistic insight into glucocorticoid‐mediated apoptotic signaling pathways (Dang et al. 2016). The Akt/ERK pathways are primarily involved in promoting cell survival and growth, with increased activation promoting physiological hypertrophy (Gallo et al. 2019). Thus, suppressed Akt/ ERK signaling diminishes protective mechanisms that normally inhibit apoptosis and hence will increase the susceptibility of cardiomyocytes to cell death. Such effects, together with enhanced catecholamine‐mediated PKA stimulation, will irrevocably shift cardiomyocytes towards apoptotic cell death.

Glucocorticoids also facilitate fibrosis in the myocardium via the mineralocorticoid receptor and the angiotensin II signaling pathways (Omori et al. 2014; Roy et al. 2009). This interaction primarily involves the modulation of angiotensin II receptor expression and subsequent signaling pathways that promote fibrotic processes in cardiac tissues. For example, although dexamethasone treatment increased AT1R expression via gene transactivator effects as a cardioprotective effect against ischemia–reperfusion injury in acute settings, this could not be replicated within a chronic setting (Xue et al. 2014). Here, chronic AT1R stimulation potentiates cardiac remodeling and fibrosis through tissue growth factor β via Smad‐dependent and non‐Smad pathways, which are further aggravated by inflammatory signaling (reviewed by Dasgupta and Zhang (2011); Frangogiannis (2020)).

In addition to their myriad intracellular effects, pro‐inflammatory cytokines such as TNF‐α can suppress estrogen production either by inhibiting the CYP17A1 enzyme (crucial for converting 17α‐hydroxyprogesterone to dehydroepiandrosterone) or by initiating apoptotic pathways in granulosa cells and oocytes (Straub et al. 2005; Shepel et al. 2014; Chaudhary et al. 2019; Kala and Nivsarkar 2016). As estrogen is known to elicit cardioprotective effects, its relative deficiency in postmenopausal women is a major risk factor for IHD and CVD. For example, an in vitro study demonstrated a negative correlation between Rho mRNA expression and estradiol levels in coronary vascular smooth muscle cells treated with angiotensin II (Hiroki et al. 2005). This suggests a potential mechanism for the increased prevalence of microvascular spasms in postmenopausal women.

### 4.3 Vascular and Cardiomyocyte Contractility

Microvascular dysfunction is defined as inadequate blood flow to meet myocardial oxygen demands, as a consequence of impaired vasodilation, increased vascular resistance, and microvascular spasms (Vancheri et al. 2020). Female patients presenting with angina and non‐obstructive IHD often show signs of microvascular dysfunction following coronary angiography, cardiac magnetic resonance imaging, or positron emission tomography procedures (Vancheri et al. 2020; Kuruvilla and Kramer 2013). Extensive research work revealed that chronic stress can substantially contribute to endothelial dysfunction and that this primarily occurs through elevated levels of glucocorticoids, catecholamines, and angiotensin‐II to result in a pro‐inflammatory milieu and impaired vascular tone (reviewed by Sher et al. (2020)). Although both are characterized by elevated oxidative stress and attenuated NO bioavailability, microvascular dysfunction specifically affects the microcirculation's ability to adequately respond to physiological demands (reviewed by Vancheri et al. (2020)). As a result of the close relationship between microvascular and endothelial dysfunction, they share common pathophysiological mechanisms. It is our opinion that, as it is involved in various cardiovascular pathologies and can be activated by stress‐related hormones and inflammatory markers, it represents a promising avenue for future research.

Upstream activators of the Rho/ROCK kinase pathway include serotonin, angiotensin‐II, norepinephrine, endothelin‐1, and pro‐inflammatory cytokines like TNF‐α (reviewed by Shimokawa and Takeshita (2005)), most of which are elevated during psychological stress conditions (Münzel et al. 2017; López‐López et al. 2016; Gang et al. 1993). Increased binding of any of the above upstream mediators to their respective G‐protein‐coupled receptors activates Rho, a small GTPase, which stimulates ROCK. The downstream molecular targets of ROCK are primarily proteins involved in inflammation and contractility. For example, increased inhibitory‐κB kinase phosphorylation via ROCK enhances its dissociation and subsequent degradation, thus allowing NFκB translocation to the nucleus to initiate transcription of pro‐inflammatory cytokines (Liu et al. 2018). These cytokines can promote the production of additional inflammatory mediators from surrounding tissues (via paracrine effects), creating a feedback loop that exacerbates local inflammation and potentiates microvascular dysfunction (Ferrari 1999).

Aside from inflammatory processes, the Rho/ROCK kinase system predominantly affects proteins involved in contraction, such as myosin phosphatases (Shimokawa and Takeshita 2005). For normal muscle contraction, a cascade of events involving calcium, calmodulin, and myosin light chain kinase leads to the phosphorylation of myosin light chains (reviewed by Shimokawa, Sunamura, and Satoh (2016)). Such phosphorylation is a critical step, as only phosphorylated myosin light chains can bind to actin and initiate the contractile process. As long as calcium levels remain elevated, myosin remains phosphorylated, and the muscle continues to contract. By inhibiting such phosphatases, increased Rho/ROCK kinase activity can sustain myosin phosphorylation, blocking relaxation and contributing to transient ischemic episodes, which prevent sufficient delivery of oxygen and nutrients to local tissues (Chang, Kamm, and Stull 2016; Fukata, Amano, and Kaibuchi 2001). Without such steps, the heart may shift away from mitochondrial oxidative phosphorylation towards anaerobic respiration under these conditions thereby resulting in impaired contractility.

**FIGURE 3:**
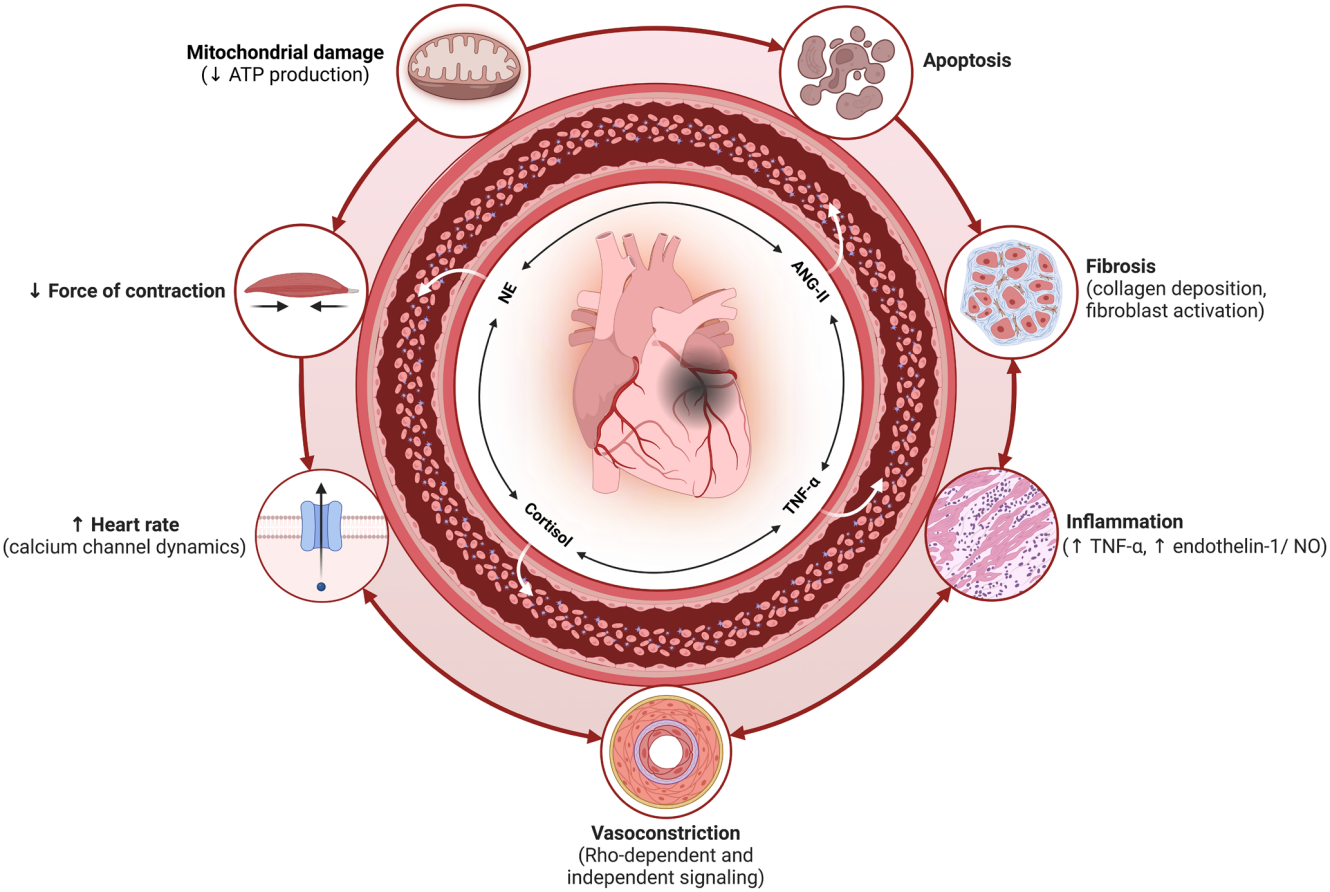
Influence of chronic stress on microvascular and cardiomyocyte dysfunction in female IHD onset and progression based on clinical and preclinical data. ANG‐II, angiotensin‐II; ATP, adenosine triphosphate; NE, norepinephrine; NO, nitric oxide; TNF‐α, tumor necrosis factor alpha. Created in BioRender.com.

Based on the available mechanisms described in the above sections, psychological stress mediators facilitate female IHD primarily via inflammatory, contractile, bioenergetic, and remodeling signaling (Figure 3). Cortisol, norepinephrine, angiotensin‐II, and pro‐inflammatory cytokines all independently and synergistically promote such pathophysiological alterations within the microvasculature and cardiomyocytes to culminate in an enhanced risk for ischemic MACE. However, most of these mechanisms are primarily derived from male in vivo preclinical studies and/or in vitro experiments. While these findings provide valuable insights, it is crucial to conduct further studies—specifically preclinical research on females—to confirm the applicability and extent of such mechanisms in the context of IHD.

## 5 Conclusion

This review aimed to elucidate the intricate relationship between chronic stress and IHD in females. By examining both clinical and preclinical evidence, we focused on the pivotal role of stress mediators in initiating cellular and molecular pathways that contribute to the development of IHD in females. To bridge the knowledge gap between preclinical research and clinical presentations, future studies should prioritize consistent and comprehensive characterization of cardiovascular contractile and bioenergetic function, as well as systemic alterations in inflammatory and stress mediators. Only by addressing such understudied areas, can we enhance our understanding of stress‐related female IHD and subsequently begin to develop more effective preventive and therapeutic strategies.

## Author Contributions

Megan Cairns conceived the idea and wrote the first draft, edited, and approved the final draft; Erna Marais and Danzil Joseph edited and approved the final draft; M. Faadiel Essop conceived the idea and wrote the first draft, edited, and approved the final draft.

## Conflicts of Interest

The authors declare no conflicts of interest.

## Data Availability

The authors have nothing to report.
